# The Clinical Application of Core-Needle Biopsy after Radiofrequency Ablation for Low-risk Papillary Thyroid Microcarcinoma: A Large Cohort of 202 Patients Study

**DOI:** 10.7150/jca.42673

**Published:** 2020-07-09

**Authors:** Lin Yan, Yukun Luo, Ying Zhang, Yaqiong Zhu, Jing Xiao, Yu Lan, Xiaoqi Tian, Qing Song, Fang Xie

**Affiliations:** 1 Medical School of Chinese PLA, No.28 Fuxing Road, Haidian District, Beijing, China.; 2 Department of Ultrasound, First Medical Center, Chinese PLA General Hospital, No.28 Fuxing Road, Haidian District, Beijing, China.; 3 Health Management Center, The Second Hospital of Dalian Medical University, No.467 Zhongshan Road, Shahekou District, Dalian, China.; 4 School of Medicine, Nankai University, No.94 Weijing Road, Nankai District, Tianjing, China.

**Keywords:** radiofrequency ablation, papillary thyroid microcarcinoma, core-needle biopsy, ultrasound.

## Abstract

**Purpose:** To evaluate the clinical application of core-needle biopsy (CNB) for low-risk papillary thyroid microcarcinoma (PTMC) after radiofrequency ablation (RFA)

**Methods:** A total of 202 patients with 211 low-risk PTMCs were included in this study. RFA procedure was used the hydrodissection technique and moving-shot technique. Patients were followed at 1, 3, 6, 12 months and every 6 months thereafter. The volume of ablation area and the volume reduction ratio (VRR) were calculated. At 3 or 6 months after RFA, CNB was performed to the central zone, the peripheral zone and surrounding thyroid parenchyma for post-ablation evaluation.

**Results:** The mean volume of tumors was 102.34±93.84 mm^3^ (range 4.19-424.10 mm^3^), which decreased significantly to 1.37±7.74 mm^3^ (range 0-73.30 mm^3^) at a mean follow-up time of 24.42±9.15 months (range 3-42 months) with a mean VRR of 99.14±4.18% (range 71.88-100%). A total of 3 ablation areas had positive CNB in the peripheral zone and underwent additional RFA. No recurrent or suspicious metastatic lymph nodes were detected

**Conclusion:** CNB is a feasible and effective evaluation for low-risk PTMC after RFA, which can detect residual cancer cells early.

## Introduction

Thyroid cancer is the most common malignancy of the endocrine system, which is responsible for 567,000 cases worldwide ranking in ninth place for incidence 1, 2. Roughly 50% of this incidence is attributable to the identification of intrathyroidal papillary thyroid microcarcinoma (PTMC) 3. PTMC is a form of papillary thyroid carcinoma (PTC) and defined by WHO as a tumor measuring 1.0 cm or less in its greatest dimension.

Surgery was generally recommended for patients with PTMC by guidelines 4, 5, but it could be associated with some permanent complications and cosmetic problems. Active surveillance was a new option recommended for patients with low-risk PTMC 4, 5. However, a patient's willingness to adopt this strategy was an important element for implementation of this protocol 6, 7. Moreover, no clinical or imaging method could reliably identify the small percent of invasive PTMC and prevent a large majority of PTMC patients from active surveillance 4, 7, 8. Thus, finding less invasive alternatives is extremely helpful and important.

Radiofrequency ablation (RFA) is a minimally invasive treatment, which has been recommended as an effective strategy for thyroid benign nodules and recurrent thyroid cancer 9. For the last few years, studies from multiple centers suggested that RFA was also an effective and safety alternative strategy for low-risk PTMC patients who were anxious about living with cancer or post-operative complications 10-13. However, its application still remained controversial. At present, the post-ablation evaluations were mainly based on ultrasound (US), which could not indicate whether all the cancer cells were completely eliminated. Although histological examination after surgery was the gold standard of diagnosis, it could not be performed to the patients who refused surgery in the first place. Biopsy like fine-needle aspiration (FNA) and core-needle biopsy (CNB), were widely used for the diagnosis of thyroid nodules 4, 14, but they were not routinely used after thermal ablation. Only a few studies with small sample size had used FNA to evaluate the ablation areas of PTMC 15, 16. However, the insufficient cellularity caused by tumor apoptosis and coagulative necrosis from thermal ablation could reduce the diagnostic accuracy of FNA for the ablation area 17, 18. Comparing with FNA, CNB had higher diagnostic accuracy and a lower non-diagnostic rate to prevent further biopsy and unnecessary diagnostic surgery 14, 18-20. To our knowledge, there were no large-scale studies about the clinical application of CNB after RFA for low-risk PTMC.

Therefore, the purpose of this study was to evaluate the clinical application of CNB for low-risk PTMC patients after RFA.

## Materials and Methods

This retrospective study was approved by the Institutional Review Board of Chinese PLA General Hospital. Written information consent was obtained from all the patients prior to CNB and RFA.

### Patients

All of the enrolled patients fulfilled the following criteria: (1) patients with PTC were confirmed by CNB; (2) tumor with a maximum diameter no larger than 10 mm; (3) absence of capsular infiltration and extrathyroidal extension; (4) no lymph nodes metastasis on US; (5) no distant metastasis; (6) patients who were unsuitable for surgery or rejected surgical treatment clearly; (7) no history of neck irradiation; (8) CNB was performed after RFA. The exclusion criteria were: (1) patients with aggressive histological PTC (e.g. tall cell, insular, columnar cell carcinoma) or benign nodule confirmed by CNB; (2) the maximum diameter of the tumor was more than 10 mm; (3) capsular infiltration and extrathyroidal extension; (4) coagulation disorder or serious heart failure/ respiratory failure/ liver failure/ renal failure; (5) conscious disturbance or neck extension disorder that could not tolerate RFA procedure; (6) cervical lymph node metastasis or distant metastasis was found; (7) contra-lateral vocal cord paralysis; (8) CNB was not performed after RFA.

From June 2016 to November 2018, 231 patients with low-risk PTMC had CNB before RFA in our institution. Among them, ablation areas of 29 patients disappeared at 3 or 6 months after RFA. The remaining 202 patients with 211 low-risk PTMCs were included in this study.

### Pre-ablation assessment

Before RFA, each tumor underwent US to evaluate the size, location, margin, shape, echogenicity, calcification and vascularity. The volume of tumor was calculated with the equations: V=πabc/6 (V is the volume, while a is the largest diameter, b and c are the other two perpendicular diameters).

US were performed using a Siemens Acuson Sequoia 512 Ultrasound System (Siemens, Mountain View, CA, USA) or a Philips iU22 Ultrasound System (Philips Healthcare, Bothell, WA) or a Mindray M9 Ultrasound System (Mindray, Shenzhen, China). CNB and RFA were all performed using a Siemens Acuson Sequoia 512 Ultrasound System.

Contrast-enhanced ultrasound (CEUS) was used to evaluate the blood supply region of the tumor before and immediately after RFA. Sulphur hexafluoride (SonoVueR, Bracco. International, Milan, Italy) was used as ultrasound contrast agent. CEUS was performed after bolus injection of SonoVue (2.4 ml), followed by a 5 ml of normal saline flush. Real-time microbubble perfusions within the tumor and surrounding tissues were observed for a minimum of 2 minutes.

### Ablation procedures

All RFA procedures were performed by an experienced US physician (Y.K.L, with more than 20 years' experience in thyroid US and interventional US). A bipolar RFA generator (CelonLabPOWER, Olympus Surgical Technologies Europe, Hamburg, Germany) and an 18-gauge bipolar RF applicator with 0.9 cm active tip were used (CelonProSurge micro 100-T09, Olympus Surgical Technologies Europe, Hamburg, Germany) in this study. Patients lay on an operating table in the supine position with the neck extended. Local anesthesia with 1% lidocaine was administered. If the distance between the tumor and critical cervical structures (trachea, cervical artery, jugular vein, esophagus and recurrent laryngeal nerve) was <5 mm, hydrodissection technique was used. Normal saline was injected using another needle (23 gauge) to separate the target tumor from critical structures in order to prevent thermal injury. RFA was performed using the trans-isthmic approach and moving-shot technique. The RFA power was 3W. If a transient hyperechoic zone did not form at the electrode tip within 5-10 seconds, the radiofrequency power was increased to 5-9 W. We enlarged the ablation area which exceeded the tumor edge (at least 3-5mm) to prevent marginal residue and recurrence 21, 22. CEUS was performed immediately after the RFA procedure to evaluate the ablation area. If any enhancement existed, a complementary ablation could be performed.

During the procedure, special attention was given to the protection of critical cervical structures in order to prevent significant complications such as hematoma or nerve injury. Each patient was observed for 1-2 hours in the hospital while any complication occurring during and immediately after ablation were carefully evaluated according to the clinical signs and symptoms.

### Post-ablation assessment

Clinical follow-ups were performed at 1, 3, 6, 12 months and every 6 months thereafter. The ablation area was evaluated by US. The development of metastatic lymph nodes was evaluated, and suspicious lesions were submitted to biopsy. The volume reduction was calculated as follows: VRR = ([initial volume - final volume] × 100)/initial volume.

### CNB procedure

At 3 or 6 months after RFA, CNB was performed. CNB procedure was performed by a disposable 1.5- or 2.2-cm excursion, 20-gauge double-action, spring-loaded needle (BARD Magumn, Bard Peripheral Vascular Inc, Tempe, USA). Because the heated ablation area could be divided into the central zone, the peripheral zone and surrounding thyroid parenchyma by thermal ablation 23-25, CNB was performed at least in the central zone and peripheral zone of the ablation area, respectively. Because the diagnostic criteria of CNB have not been standardized, the CNB results were categorized into the same six categories as those for FNA cytology, according to the Bethesda System for Reporting Thyroid Cytopathology. Bethesda VI was defined as positive CNB, Bethesda I, III and V as inconclusive results, and Bethesda II as negative CNB.

### Statistical analysis

Statistical analysis was performed using the SPSS statistical software (Version 25.0). Continuous data were expressed as mean± SD (range). Wilcoxon signed rank tests were used to compare changes in the mean volume before RFA and at each follow-up point after RFA. A difference with *P* < 0.05 was considered as statistically significant.

## Results

Clinical characteristics of patients are presented in Table 1. Among the 202 patients (including 152 females and 50 males), 194 patients had one tumor, 7 patients had two tumors and 1 patient had 3 tumors. The mean initial diameter was 5.35±1.63 mm (range, 2.00-9.33 mm) and the mean initial volume was 102.34±93.84 mm^3^ (range, 4.19-424.10 mm^3^). The mean age of patients was 42.79±10.13 years (range, 20-74 years) and the mean follow-up time of 24.42±9.15 months (range 3-42 months). There were 114 tumors located in the right lobe, 92 in the left lobe and 5 in the isthmus.

The mean power was 4.55±1.16 W (range 3-9 W). The mean RFA time was 153.81±88.11 s (range 34-530 s) and the mean energy was 767.68±461.78 J (range 160.00-2790.00 J).

**Figure 1:**
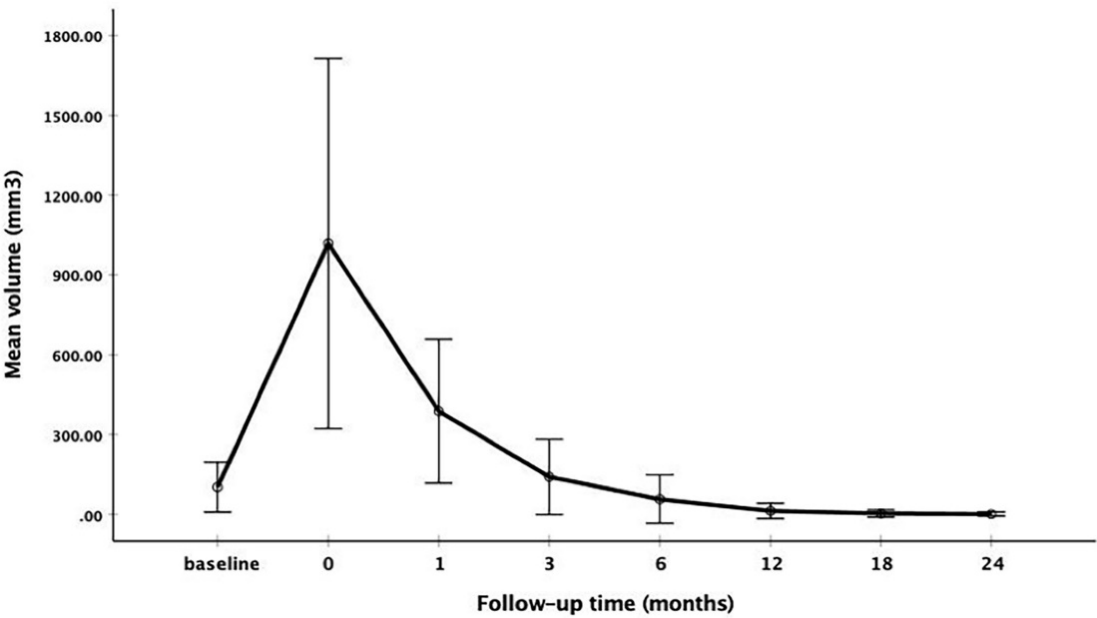
The changes of mean volume at each follow-up point

**Figure 2:**
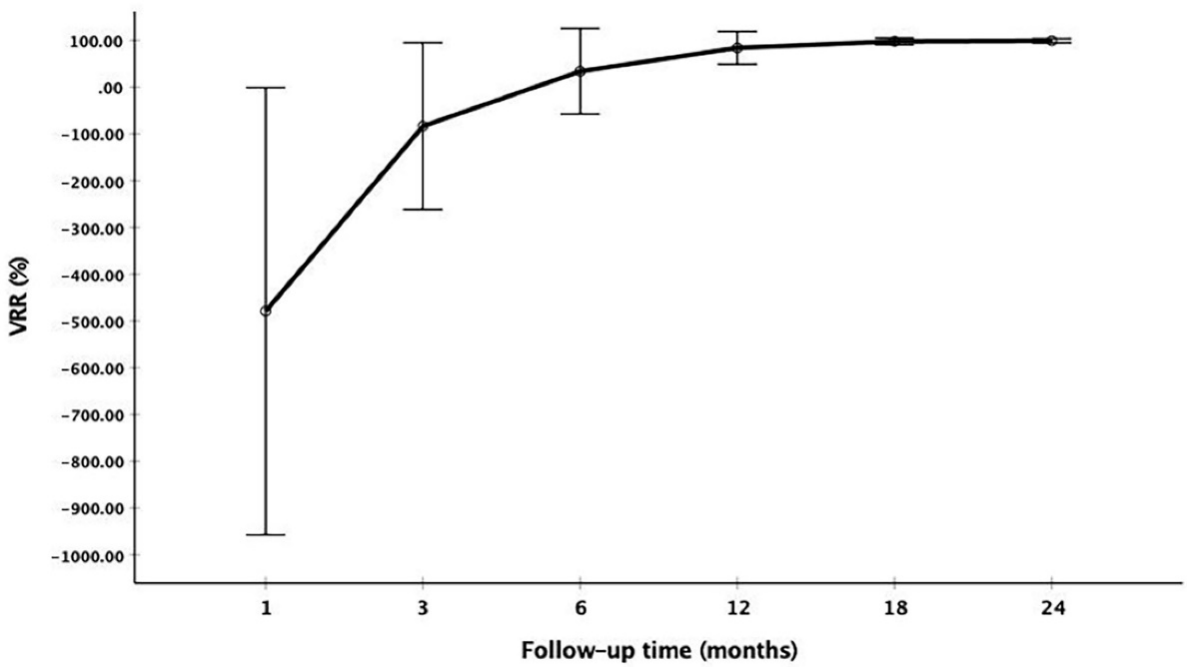
The changes of VRR at each follow-up point

**Figure 3:**
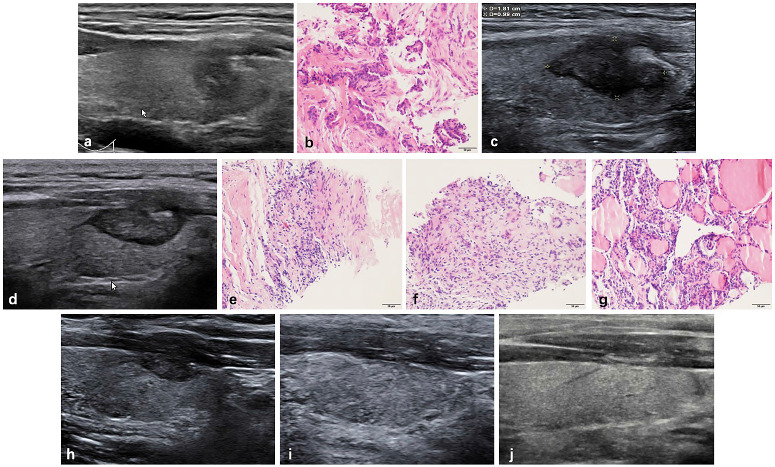
Radiofrequency ablation (RFA) treatment and follow-up of a 38-year-old male with a low-risk PTMC located in the left thyroid. (a, b) Before RFA, PTMC was diagnosed by CNB with an initial volume of 175.92 mm^3^(arrow). Ablation area(arrow) was 1810.55 mm^3^ immediately after RFA. (c) At 1 months after RFA, the ablation area(arrow) was 1130.94 mm^3^. (d) At 3 months after RFA, the ablation area(arrow) was 351.85 mm^3^(d). Post-ablation pathology showed degenerated and necrotic follicular epithelia with lymphocyte infiltration in the central zone(e), interstitial fibrous tissue hyperplasia with lymphocyte infiltration and multinucleated giant cell reaction in the peripheral zone(f) and follicular epithelia hyperplasia with lymphocyte infiltration in the surrounding thyroid parenchyma(g). (h) At 6 months after RFA, the ablation area(arrow) was 131.94 mm^3^. (i) At 12 months after RFA, the ablation area was completely disappeared, and remained to be stable at 18 months after RFA (j).

The changes of mean volume and VRR at each follow-up point are shown in Table 2. Due to the enlarge ablation, the mean volume of the ablation areas at 1 and 3 months were significantly larger than the initial volume (*P*<0.001). However, 3 months after RFA, the mean volume was gradually decreased (Figure 1 and Figure 2). The mean volume at each follow-up point was as follows: 387.84±270.43 mm^3^ (range 8.38-1524.67 mm^3^) at 1 months, 141.68±141.39 mm^3^ (range 1.05-1123.09 mm^3^) at 3 months, 57.27±91.16 mm^3^ (range 0-791.66 mm^3^) at 6 months, 13.36±29.28 mm^3^ (range 0-209.43 mm^3^) at 12 months, 3.51±13.99 mm^3^ (range 0-128.28 mm^3^) at 18 months and 1.37±7.74 mm^3^ (range 0-73.30 mm^3^) at 24 months. At last follow-up point, the mean VRR was 99.14±4.18% (range 71.88-100%). A total of 139 ablation areas (65.88%) completely disappeared. The numbers of completely disappearance were 39(18.5%), 71(33.64%), 16(7.58%) and 13(6.16%) at 6, 12, 18 months and 24 months after RFA, respectively (Figure 3).

During the follow-up, CNB was performed at 3 or 6 months after RFA. Among CNB for 211 ablation areas, 75.83% (160/211) were performed in 3 zones and 24.17% (51/211) in two zones. The mean volume of ablation areas was 118.37±131.84 mm^3^ (1.05-1123.09 mm^3^) when CNB was performed after RFA. A total of 3 ablation areas had positive CNB which were all Bethesda VI in the peripheral zone and then underwent additional RFA. The changes of volume and VRR are showed in Table 3.

All the patients were well tolerable to RFA and CNB procedure. No recurrent or suspicious metastatic lymph nodes were detected.

## Discussion

This study demonstrated the clinical application of CNB for low-risk PTMC after RFA. During a mean follow-up time of 24.42±9.14 months, the mean VRR of low-risk PTMC was 99.14±4.18%. No recurrent or suspicious metastatic lymph nodes were detected. A total of 3 ablation areas were diagnosed to have residual cancer by CNB and had additional RFA. All the patients were tolerant to the CNB procedure and no complications occurred. These results indicated that CNB could be used to evaluate the efficacy of RFA for low-risk PTMC.

For the last few years, several studies from multiple centers suggested that RFA was an effective and safe alternative strategy for low-risk PTMC 10-13. After ablation, the volume reduction of ablation area was significant, and some tumors even disappeared in the follow-up. However, recently, Ma et al. 26 reported surgical confirmation of residual cancer in 11 PTC patients and 1 PTMC patient after previous ablation and suggested thermal ablation may result in incomplete treatment. Therefore, it is necessary to confirm the complete ablation of the target tumor to achieve the curative purpose. The Chinese expert consensus on thermal ablation for thyroid benign nodules, microcarcinoma and metastatic cervical lymph recommended that the efficacy of thermal ablation could be evaluated by biopsy at early stage of follow-up 27. Two studies had used FNA to evaluate the ablation areas of PTMC at 1, 6 and 12 months after thermal ablation 15, 16. Zhou et al 15 reported FNA was performed to the ablation area at 1,6 and 12 months after laser ablation (LA) for 30 low-risk PTMCs. In another study about LA for 64 low-risk PTMCs, FNA was performed in 3 zones of ablation areas at 1,6 and 12 months 16. In our previous study, CNB was performed to the ablation areas at 3 months after RFA, however, the follow-up time was only 7.8 months 10. In addition, CNB was also used to evaluate the ablation areas of recurrent thyroid cancer and benign nodules 28-30. There were three reasons why we chose CNB instead of FNA to evaluate the ablation areas. First, although FNA was recommended for the diagnosis of thyroid nodules, it had limitations of low diagnostic accuracy and a high false-negative rate 20, 31-34. The insufficient cellularity caused by tumor apoptosis and coagulative necrosis from thermal ablation could also reduce the diagnostic accuracy of FNA 17, 18. Second, Comparing with FNA, CNB had higher diagnostic accuracy and a lower non-diagnostic rate to prevent further biopsy and unnecessary diagnostic surgery 14, 18-20. Third, CNB was safe, well-tolerated and associated with a low incidence of complications when performed by experienced operators 14. We already had the experience of CNB for the ablation area of low-risk PTMC 10, which could also reduce incidence of complications.

In this study, 3 ablation areas had positive CNB in the peripheral zone. The explanation might be that during the RFA procedure, the peripheral zone had a temperature between 41°C and 45°C, which only cause sublethal and indirect damage leading to apoptosis or recovering from reversible injury 17. Positive CNB suggested some cancer cells still existed, which might recover from the sublethal damage or not be completely eliminated by RFA. It was important to note that all the positive CNB was found in the peripheral zone, which suggested this zone was critical for complete ablation of low-risk PTMC. Similarly, several studies found that recurrent benign nodule after RFA showed regrowth in the peripheral zone 35-37. However, unlike benign nodule, the primary purpose of RFA for low-risk PTMC was not to reduce symptom of pressure or solve cosmetic problem, but to obtain complete ablation. Therefore, the peripheral zone of low-risk PTMC should be ablated carefully and completely.

At present, the post-ablation evaluations for low-risk PTMC and benign nodule were both based on US. In this study, a total of 3 ablation areas had positive CNB, but the volumes were decreased. This rather contradictory result indicated that volume reduction was not equal to the disappearance of cancer cells. Only using US to evaluate the ablation area of low-risk PTMC was not enough, because it could neither provide diagnostic information about complete treatment nor detect residual cancer cells in the early period of follow-up.

This study including 202 patients with 211 low-risk PTMCs, has been not only the largest cohort of low-risk PTMC treated by thermal ablation thus far, but also the first study to demonstrate the value of CNB after ablation. It showed several advantages of CNB performed after ablation. First, CNB could provide a histological information on whether all the cancer cells were completely eliminated after ablation for low-risk PTMC. Second, compared with other evaluations by US, CNB could detect residual cancer cells earlier. Therefore, additional RFA could be performed in time to achieve complete ablation. Third, unlike previous studies which applied FNA three times after ablation, CNB in this study was only performed once after RFA. It could not only prevent multiple biopsies in multiple follow-up visits, but also avoid complications after biopsy.

There are some limitations in this study. First, it was a single-center retrospective study and the follow-up time was relatively short. Second, since we could not perform surgery on the ablation areas, the diagnostic performance of CNB could not be evaluated. However, in this study, 75.83% of CNB were performed in 3 zones of the ablation area and 24.17% in 2 zones, which could improve the diagnostic accuracy. Third, the sensitivity of US to detect central metastatic lymph nodes and multifocality was low, and their presences could not be completely excluded.

In conclusion, CNB is a feasible and effective evaluation for low-risk PTMC after RFA, which can detect residual cancer cells early. By CNB, RFA can be used as an efficacy and safety alternative for patients with low-risk PTMC.

## Figures and Tables

**Table 1:** Clinical characteristics of patients before RFA.

Characteristics	Data
Age	42.79±10.13 (range 20-74)
Sex (female/male)	152/50 (75.25%/24.75%)
Follow-up time (months)	24.42±9.15 (range 3-42)
Mean diameter (mm)	5.35±1.63 (range 2.00-9.33)
Volume (mm^3^)	102.34±93.84 (range 4.19-424.10)
No. of patients	202
No. of tumors	211
One tumor	194(91.94%)
Two tumors	14(6.64%)
Three tumors	3(1.42%)
Location	
right lobe	114(54.03%)
left lobe	92(43.60%)
isthmus	5(2.37%)

**Table 2:** Changes of the volume and VRR after RFA at each follow-up.

Time	Volume of ablation area (mm^3^)		VRR (%)		*p* value (Vs initial volume)
	Mean volume	range	Mean VRR	range	
initial	102.34±93.84	4.19-424.10	NA		-
After RFA	1018.17±696.03	146.60-6217.55	NA		<0.001
1 month	387.84±270.43	8.38-1524.67	-479.28±478.53	-2477.14-55.56	<0.001
3 months	141.68±141.39	1.05-1123.09	-83.51±178.54	-1013.75-94.44	<0.001
6 months	57.27±91.16	0-791.66	34.00±91.67	-462.50-100	<0.001
12 months	13.36±29.28	0-209.43	84.01±34.91	-157.81-100	<0.001
18 months	3.51±13.99	0-128.28	97.81±6.99	65.97-100	<0.001
24 months	1.37±7.74	0-73.30	99.14±4.18	71.88-100	<0.001

NA: not applicable

**Table 3:** Volume changes of the ablation areas with positive CNB.

No. of tumor	Volume (mm^3^)						VRR (%)		
	Initial	After RFA	1 month	3 months	6 months		1 month	3 months	6 months
1	175.92	2089.10	560.76	167.55	109.95		-218.75	4.76	37.5
2	424.10	2457.18	678.56	329.86	301.58		-60.00	22.22	28.89
3	234.57	980.15	322.53	205.24	131.94		-37.50	12.50	43.75

## Abbreviations

Core-needle biopsy: CNB
RFA: radiofrequency ablation
PTMC: papillary thyroid microcarcinoma
PTC: papillary thyroid carcinoma
US: ultrasound
VRR: volume reduction ratio
FNA: fine-needle aspiration
CEUS: contrast-enhanced ultrasound
